# Breastfeeding and respiratory, ear and gastro-intestinal infections, in children, under the age of one year, admitted through the paediatric emergency departments of five hospitals

**DOI:** 10.3389/fped.2022.1053473

**Published:** 2023-02-15

**Authors:** Bernard Branger, Amaïa Bainier, Laureen Martin, Estelle Darviot, Aude Forgeron, Laurent Sarthou, Anne-Claire Wagner, Thomas Blanchais, Thomas Brigly, Françoise Troussier

**Affiliations:** ^1^ Department of Pediatrics, Epidemiologist, Nantes, France; ^2^ Department of Pediatrics, Centre Hospitalier Universitaire, Angers, France; ^3^ Department of Pediatrics, Centre Hospitalier Robert Bisson, Lisieux, France; ^4^ Centre Hospitalier Universitaire, Angers, France; ^5^ Department of Pediatrics, Centre Hospitalier, Le Mans, France; ^6^ Department of Pediatrics, Centre Hospitalier, Cholet, France; ^7^ Department of Pediatrics, Centre Hospitalier, Saint Nazaire, France; ^8^ Paediatrician, Centre Hospitalier, Boulevard Stéphane Moreau, La Roche-sur-Yon, Nantes, France; ^9^ Department of Pediatrics, Nantes, France; ^10^ Department of Pediatrics, Saint-Jean-de-Védas, France

**Keywords:** breastfeeding, respiratory infections, gastro-intestinal infections, multicentric study, protective effect

## Abstract

**Background:**

Breastfeeding is a protective factor against respiratory and intestinal infections in developing countries. In developed countries, proof of this protection is more difficult to show. The objective of the study is to compare the proportion of children breastfed during their first year in groups of children with infectious pathologies supposedly prevented by breastfeeding and children free of these infectious pathologies.

**Method:**

Questionnaires about diet, socio-demographic data and the motive for consultation were given to the parents upon arrival in the paediatric emergency departments of 5 hospitals located in Pays de Loire (France) in 2018 and 2019. Children with lower respiratory tract infections, acute gastroenteritis and acute otitis media were included in the case group (A), children admitted for other reasons were included in the same control group (B). Breastfeeding was classified as exclusive or partial.

**Results:**

During the study period, 741 infants were included, of which 266 (35.9%) in group A. In this group, children were significantly less likely to have been breastfed at the time of admission than children in group B: for example, for children under 6 months, 23.3% were currently breastfed in group A, vs. 36.6% (weaned BF or formula diet) in group B [OR = 0.53 (0.34–0.82); *p* = 0.004]. Similar results were found at 9 and 12 months. After taking into account the age of the patients, the same results were confirmed with an aOR = 0.60 (0.38–0.94) (*p* = 0.02) at 6 months, but with when considering six variables six variables, aOR was not significative aOR = 0.65 (0.40–1.05); *p* = 0.08), meaning that factors such as the childcare out of home, socio-professional categories, and the pacifier decrease the protective effect of breastfeeding. Sensitivity analyses (age-matching, analysis by type of infection) showed the same protection effect provided by breastfeeding when it was pursued for at least 6 months and also that the protective effect of breastfeeding is especially true against gastro-enteritis.

**Conclusion:**

Breastfeeding is a protective factor against respiratory, gastrointestinal and ear infections when pursued at least 6 months after birth. Other factors such as collective childcare, pacifiers and low parental professional status can reduce the protective effect of breastfeeding.

## Introduction

In France, the prevalence of breastfeeding (BF) is low (70% according to the ELFE study (1, 2) and 74% according to the EPIFANE study (3)) and is getting even lower, only 66.7% of mothers start breastfeeding after birth according to a perinatal survey carried out in 2016.

The period during which babies are breastfed after birth is short, between 15 and 17 weeks (4, 5). In the Pays de Loire region (like the western part of France as a whole) the proportion on mothers breastfeeding after giving birth is even lower, 57.8% in 2019 and a median period of being breastfed was 15 weeks in 2012 (6). A literature review (7) and the 2003 world health organization (WHO) recommendations (8) show that, in developing countries, the benefits of breastfeeding when it came to preventing acute gastroenteritis, respiratory tract and ENT infections are widely recognized. However, in developed countries, these benefits, when compared to newborns fed with formula, are harder to prove; there are very few studies and that have only recently been published about the effects of breastfeeding. Over the last 10 years, four studies have been published: (i) a Swiss cohort study with 436 children in 2016 (9) showed that BF reduced the incidence and the severity score of respiratory symptoms during the first six months after birth, mainly the first 27 weeks vie (relative risk (RR) = 0.70; 95% confidence interval (95%CI) = 0.55–0.88), (ii) a case control study involving 273 Italian children in 2017 (10) showed that risk factors for respiratory tract infections in hospitalized children when compared to non-infected control patients were having a sibling, being exposed to passive smoking and having been breastfed for less than 3 months [risk being reduced if > 3 months: odds ratio (OR) = 0.5; 95%CI = 0.3–0.9], (iii) a third, less recent study, carried out in Greece in 2010 (11) showed that BF protected from respiratory tract infections (adjusted odds ratio (aOR) = 0.58 (0.36–0.92)) and oral thrush for 6 months when taking confounding variables into account as well as helping to reduce hospital admissions for these conditions, (iiii) a study in Denmark published in 2020 showed a reduced risk of hospitalization in a cohort of 1.087 children depending on the duration of breastfeeding. For each additional month of breastfeeding, a 5% reduction in hospitalization was observed (12). In France, to date, no research studies have been conducted on this subject.

Knowing this, the objective of this study is to estimate the proportion of children breastfed affected by conditions supposedly prevented by BF (infected children) and compare it to the proportion of breastfed children having other conditions without any known correlation with BF (non-infected children).

## Materials and methods

We conducted a non-interventional research study between 2018 and 2020. The study was approved by Angers university hospital's ethics committee, reference number 2019–93. Data was collected, in addition to the university hospital in Angers, from four other hospitals located in the Pays de Loire region, western France: Saint Nazaire, Cholet, le Roche-sur-Yon and Le Mans. The patients included were from the paediatric emergency departments, were 0–1 year old at the time of inclusion (12 months or 52 weeks) and born at term (gestational age ≥37 weeks of amenorrhea). The periods during which inclusions took place were autumn, winter and spring in order to include children that were considered infected: September 2018 through to April 2019 and September 2019 through to March 2020. Parental consent was obtained upon inclusion.

Each group was defined as follows: (*) Group A: children having had a consultation in the paediatric emergency department for an acute respiratory tract infection (bronchitis, bronchiolitis, pneumonia …), an ENT infection (otitis, sore throat …) or acute gastroenteritis, (**) Group B: children having had a consultation or admitted *via* the paediatric emergency for other conditions (traumatic injuries, other respiratory infections …).

The patient's diet was categorized at the time of inclusion in the emergency department (3, 4): (i) exclusively breastfed (EBF) (or predominantly): the patient was fed thanks to breast milk, without any other liquids (water, sweetened water, infusions, fruit juice …) or solid food; only oral rehydration solution, medications or vitamins/minerals (in liquid form: syrup or drops) were permitted, (ii) partially breastfed (PBF): the patient was fed with breast milk and formula milk made essentially from cow's milk, other types of liquid intake were permitted (water, sweetened water, infusions, fruit juice …) (iii) formula diet (FD): the child was fed exclusively with formula milk. Dietary diversification was defined as a regular consumption of at least one solid or semi-solid food, cow or other animal's milk or plantbased “milks”. Children considered EBF were considered as such until they were fully weaned, even if they'd started a diversified diet. To be considered a regular consumption, a type of food must have been eaten by the child and not simply have been tasted occasionally. Fully weaned was defined as breastfeeding having completely stopped at a specific date leading to an EBF or PBF period (in weeks) for each patient. For the analysis, multiple groups were pooled together depending on their dietary status at the time of inclusion: (1) EBF or PBF ongoing or weaned vs. FD, (2) EBF or PBF ongoing vs. fully weaned or FD, (3) EBF vs. PBF or fully weaned or FD. The breastfeeding period was defined as the period between birth and the child being fully weaned.

Other characteristics collected during the study were: date of birth and date of consultation in the emergency department, child's age, gender, feeding status at birth (EBF, PBF or FD), birth weight, type of birth, pre-existing conditions, social class based on parental occupation, siblings and their feeding status', parental tobacco consumption, pacifiers, type of childcare, feeding status upon arrival in the emergency department (EBF, PBF, FD) as well as when they were fully weaned and their diet diversified, if applicable.

### Statistical methods

The reasoning behind the sample size was as follows: the estimated rate of breastfeeding (EBF + PBF) at birth was 55% and 30% at 4 months (6). The expected differential of breastfeeding rates between infected and non-infected children was 10%, which corresponds to 35% for breastfed children vs. 25% for children fed with formula at the age of 4 months. Based on this with a alpha risk at 0.05, a power at 80% and two tails hypothesis, one patient in group A for every two in group B, the number of subjects required were 250 and 500, i.e., 750 in total.

Qualitative data was expressed in percentages with a 95% confidence interval (95CI), quantitative data using averages ± mean standard error, or median value with maximum and minimum values.

Comparisons were made using chi-square tests for proportions and Student tests as well as ANOVA for averages. The univariate analysis results are expressed in crude odds ratios, with 95% confidence interval, in order to determine the strength of the association between breastfeeding and infected or non-infected children. An OR < 1 means that infected children were less frequently breastfed than the ones that weren't considered infected.

Numerous confounding factors appeared when comparing the rates of breastfeeding between infected and non-infected children, such as the child's age, social status (based on the parents' occupation), whether parents occupied a full or part-time job, whether pacifiers were used and, if so, all day or only during night time, family or personal history of allergies and parental tobacco consumption.

A multivariate analysis was carried out using logistic regression in order to adjust the OR (aOR) and take into account the confounding factors. The variables with *p* < 0.20, plus the “sex” variable, have been introduced into the complete models with two phases: an adjustment exclusively on age and an adjustment on 6 variables: the age of the child, the socio-professional category of the parents, the parents or sibling allergies, the type of childcare (home vs. others), the use of a pacifier, the gender of the child. The variable “full-time job mother or father” being correlated with socio-professional categories, and “diversification” with food status. SPSS 22.0 software was used for the statistical analysis. Three sensitivity studies were carried out: (1) Group A was expanded in order to include other infections such as rhino pharyngitis, laryngitis and sore throats which are not known to be prevented by breastfeeding, (2) To take into account the effect of the patients' age on the rate of breastfeeding, patients from group A were matched based on their age with patients from group B (conditional logisitic regression models were used thanks to the SPSS 22.0 software and the “R” software for matching); (3) A specific and separate study was carried out on patients with bronchitis and gastroenteritis.

## Results

### Study population characteristics

**Figure 1:**
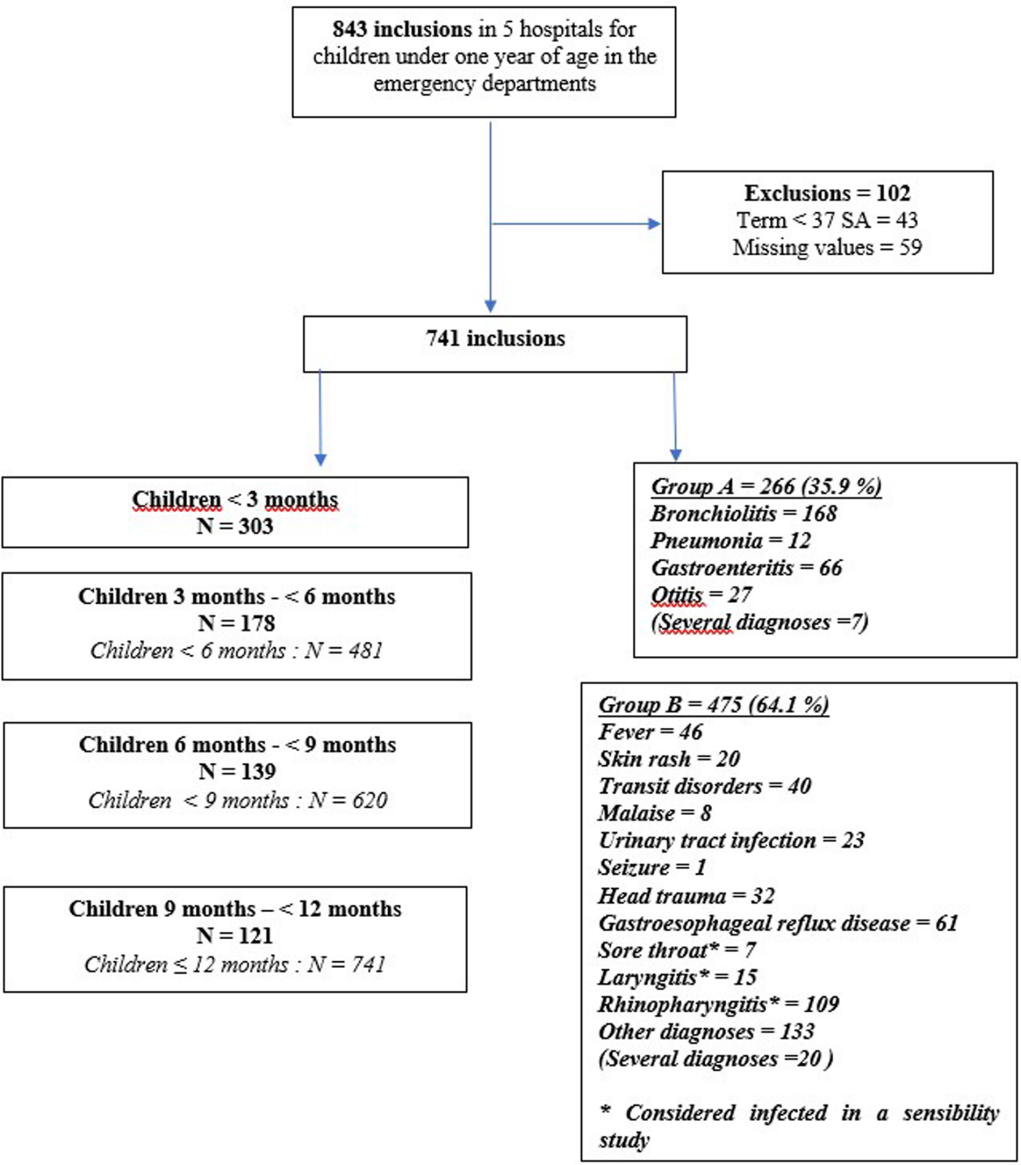
Flow-chart.

We collected data from 843 infants that came to the paediatric emergency department in the participating hospitals; 741 of these patients were included in our study (Figure 1). The main reason behind infants not being included was a gestational age at birth below 37 weeks of amenorrhea, and the missing values. The characteristics of the children are detailed in Table 1. The median consultation age was 4.7 months. Amongst the patients included, 28.2% were still breastfed at the time of consultation, of which 19.0% were exclusively.

**Table 1:** Description of the child population.

Variables	*N* (%) mean ± mean standard error (minimum – maximum) or median (interquartile range) *n* = 741
Children data	
Age at admission (month)	3.6 (1.7–7.5)
Gender (boys)	396 (53.4)
Birth weight (g)	3 308 ± 18 (1 710–5 560)
Term (AW*)	39.0 (38.0–40.0)
Cesarean (%)	135 (18.2)
Twins (%)	20 (2.7)
Weight at admission (kg)	6.4 ± 0.08 (2.2–13.1)
Food at birth (%)	
EBF**	294 (39.7)
PBF***	87 (11.7)
Total BF****	*371 (51.4)*
FD*****	360 (48.6)
Food at admission (%)	
EBF	141 (19.0)
PBF	68 (9.2)
*Total BF*	*209* (*28.2)*
FD	532 (71.8)
Duration BF for breastfed children (months)	
EBF	1.9 ± 0.2 (0.03–12)
PBF	2.7 ± 0.2 (0.13–12)
Diversification started (%)	307 (41.4)
Age of diversification (months)	4.5 (4.0–5.3)
Family context	
Age of the mother (years)	29.7 ± 0.19 (16–50)
Executive job mother	154 (20.8)
Executive job father	232 (31.3)
Executive job mother or father	282 (38.1)
Risk factors of infection	
Sibling ≥1	458 (61.8)
Parents and / or sibling allergies	385 (52.0)
Smoking mother	146 (19.7)
Smoking father	248 (33.5)
Smoking father or mother	291 (39.3)
Pacifier (%)	
Often	263 (35.5)
Sometimes	318 (42.9)
*Total using pacifier*	*581* (*78.4)*
Never	160 (21.6)
Childcare (%)	
Home	456 (61.5)
Other sites and collective childcare	285 (38.5)

* AW : amenorrhea weeks

** EBF : Exclusively breastfed

*** PBF : Partially breastfed

**** BF : Breastfed

***** FD : Formula diet

### Infections and feeding status at the time of consultation

Table 2 (univariate analysis) shows that patients in group A were less frequently breastfed (BF vs. FD) at the time of consultation than those in group B [20.7% vs. 32.4%; OR = 0.54 (0.38–0.77); *p* = 0.002] whereas, at birth, with regard to breastfeeding, there was no significant difference between the two groups. However, there were differences between the two groups: patients in group A were older than patients in group B (5.5 months vs. 4.2 months), weighed more at the time of consultation, had more frequently a first degree family history of allergies, used pacifiers more often (either all day or only at night) and childcare was more likely to be collective. The parents of these patients occupied more frequently executive roles and were in full-time employment. These differences were potentially confounding factors, especially the age difference.

**Table 2:** Comparison between infected children and non-infected children (univariate analysis).

Variables	Group A^a^ (*N* = 266)	Group B^a^ (*N* = 475)	Crude Odds Ratios^d^ (95% CI)	*p*
Admission				
Age (months)	5.56 ± 0.21	4.26 ± 0.16		<10^−5^
Gender (% boys)	154 (57.9)	242 (50.9)	1.32 (0.98–1.79)	0.07
Weight (kg)	6.88 ± 0.13	6.15 ± 0.09		10^−6^
Type of food^b^ at admission				
EBF	32 (12.0)	109 (22.9)		
PBF	23 (8.7)	45 (9.5)		
*Total BF (EBF* *+* *PBF)*	*55 (20.7)*	*154 (32.4)*	0.54 (0.38–0.77)	0.002
FD	211 (79.3)	321 (67.6)	1	
Diversification started (%)	130 (48.9)	177 (37.3)	1.61 (1.19–2.18)	0.001
Family context				
SPC^c^ executive or middle level – mother or father (%)	125 (47.0)	157 (33.1)	1.79 (1.32–2.44)	0.0001
Full-time job mother (%)	106 (39.8)	150 (31.6)	1.43 (1.05–1.96)	0.02
Full-time job father (%)	238 (89.5)	377 (79.4)	2.21 (1.41–3.47)	0.0001
Risk of infection				
Sibling ≥1 (%)	171 (64.3)	287 (60.4)	1.18 (0.86–1.61)	0.30
Parents or sibling allergies (%)	152 (57.1)	233 (49.0)	1.38 (1.02–1.89)	0.03
Smoking mother (%)	54 (20.3)	92 (19.4)	1.06 (0.73–1.54)	0.76
Smoking father (%)	86 (32.3)	162 (34.1)	0.92 (0.67–1.27)	0.62
Smoking mother or father (%)	101 (38.0)	190 (40.0)	0.92 (0.67–1.25)	0.59
Pacifier often or to sleep (%)	222 (83.5)	359 (75.6)	1.63 (1.10–2.40)	0.012
Childcare at home (%)	140 (52.6)	316 (66.5)	0.56 (0.41–0.76)	0.0001

^a^ Group A: infected children, affected by bronchiolitis, pneumonia, gastroenteritis and otitis; Group B: noninfected children, affected by other pathologies.

^b^ EBF: Exclusively breastfed, PBF: Partially breastfed, BF: Breastfed, FD: Formula diet.

^c^ SPC: Socio-professional category.

^d^ *OR < 1 means that Group A children are breastfed less often*

Furthermore, there was an association between pacifier use and breastfeeding. Using a pacifier, parents occupying a senior job and collective childcare seemed to increase the risk of infection. These factors that caused gastrointestinal and respiratory tract infections could antagonise the protective role of BF. The Table 3 shows the crude ORs according to age categories and the three types of diet: the benefit of breastfeeding appeared from 6 months for current BF or current EBF, but not for the current or weaned BF. For example, before the age of 6 months, the OR for ongoing BF was 0.53 (0.34–0.82; *p* = 0.004) and the OR for EBF was 0.59 (0.36–0.95); *p* = 0.023). Before the age of 3 months, children in group A were less BF than in group B, but there was no difference if we only compared patients having been EBF. The univariate analysis showed no difference when comparing ongoing BF and fully weaned as a whole with other diets.

**Table 3:** Comparison of food since birthday between infected children and non-infected children by age-group - Univariate analysis.

Type of food^a^	Group A^b^	Group B^b^	Crude OR (IC 95%)	*p*
Age < 3-month-old (<13 weeks)	*N* = 77	*N* = 226		
Current BF or weaned BF vs. FD	36 (46.8)	117 (51.8)	0.82 (0.49–1.37)	0.45
Current BF vs. weaned BF or FD	21 (27.3)	91 (40.3)	0.56 (0.31–0.98)	0.04
Current EBF vs. others	14 (18.2)	64 (28.3)	0.56 (0.29–1.07)	0.08
Age < 6-month-old (<26 weeks)	*N* = 150	*N* = 331		
Current BF or weaned BF vs. FD	69 (45.3)	168 (50.8)	0.80 (0.55–1.18)	0.27
Current BF vs. weaned BF or FD	35 (23.3)	121 (36.6)	0.53 (0.34–0.82)	0.004
Current EBF vs. others	27 (18.0)	90 (27.2)	0.59 (0.36–0.95)	0.03
Age < 9-month-old (<39 weeks)	*N* = 211	*N* = 409		
Current BF or weaned BF vs. FD	100 (47.4)	208 (50.9)	0.87 (0.62–1.21)	0.41
Current BF vs. weaned BF or FD	44 (20.9)	136 (33.3)	0.53 (0.36–0.78)	0.001
Current EBF vs. others	30 (14.2)	101 (24.7)	0.50 (0.32–0.79)	0.002
Age <12-month-old (≤52 weeks)	*N* = 266	*N* = 475		
Current BF or weaned BF vs. FD	133 (50.0)	243 (51.1)	0.96 (0.71–1.29)	0.76
Current BF vs. weaned BF or FD	51 (19.2)	150 (31.6)	0.51 (0.36–0.74)	0.0002
Current EBF vs. others	37 (13.9)	116 (24.4)	0.50 (0.33–0.75)	0.001

^a^ EBF, Exclusively breastfed; PBF, Partially breastfed; BF, Breastfed; FD, Formula diet.

^b^ Group A, infected children, affected by bronchiolitis, pneumonia, gastroenteritis and otitis; Group B, non-infected children, affected by other pathologies.

→* OR < 1 means* *that Group A children are breastfed less often.*

The multivariate analysis shown in Table 4 takes into account either age only or a set of 6 variables: age of the child, socio-professional category of the parents, family allergies, type of childcare (home vs. others), use of a pacifier, and the gender of the child. After adjustment on age of the child, the aOR remained significant from 6 months for currently BF and EBF, but with adjustment using six variables, the aORs were no longer significant at 6 months or 9 months, but only at 12 months. These results showed that adjusment factors decrease the protective effect of breastfeeding, especially family allergies [aOR = 1.40 (1.02–1.92); *p* = 0.038], favored socio-professional parents [aOR = 1.85 (1.34–2.55); *p* = 0.001], and use of a pacifier [aOR = 1.72 (1.13–2.61); *p* = 0.012], three factors that are positively associated with the risk of infection.

**Table 4:** Comparison of food since birthday between infected children and non-infected children by age-group - Multivariate analysis (multiple logistic regression).

Type of food^a^	Adjustment on age			Ajustment on 6 variables^b^		
	aOR	IC 95%	*p*	aOR	IC 95%	*p*
Age <3-months (<13-weeks-old)						
Current BF or weaned BF vs. FD	0.85	0.51–1.43	0.54	0.96	0.55–1.67	0.88
Current BF vs. weaned BF + FD	0.65	0.37–1.12	0.12	0.69	0.38–1.26	0.23
Current EBF vs. others	0.69	0.37–1.28	0.24	0.76	0.39–1.49	0.43
Age <6-months (<26-weeks-old)						
Current BF or weaned BF vs. FD	0.82	0.55–1.22	0.33	0.89	0.58–1.36	0.59
Current BF vs. weaned BF + FD	0.60	0.38–0.94	0.024	0.65	0.40–1.05	0.08
Current EBF vs. others	0.60	0.37–0.99	0.04	0.71	0.41–1.20	0.20
Age <9-months (<39-weeks-old)						
Current BF or weaned BF vs. FD	0.87	0.62–1.21	0.41	0.93	0.65–1.32	0.68
Current BF vs. weaned BF + FD	0.59	0.40–0.89	0.011	0.68	0.45–1.05	0.08
Current EBF vs. others	0.57	0.36–0.89	0.015	0.67	0.41–1.08	0.10
Age <12-months						
Current BF or weaned BF vs. FD	0.92	0.68–1.25	0.59	0.99	0.72–1.37	0.95
Current BF vs. weaned BF + FD	0.58	0.40–0.84	0.004	0.66	0.44–0.98	0.038
Current EBF vs. others	0.54	0.36–0.82	0.004	0.61	0.39–0.95	0.03

^a^ EBF, Exclusively breastfed; PBF, Partially breastfed; BF, Breastfed; FD, Formula diet.

^b^ Adjustment on the age of the child, the socio-professional category of the parents, the parents or sibling allergies, the type of childcare (home vs. others), the use of a pacifier, the gender of the child.

*= OR < 1 means that Group A children are breastfed less often*.

### Sensitivity studies

Infected and non-infected children were matched according to their age at the time of their consultation in the emergency department, 266 infected children were matched with 266 non-infected children. Despite comparing groups after age matching, infections and lower rates of BF were still associated [20.1% vs. 30%; OR = 0.60 (0.40–0.88); *p* = 0.016], even after adjusting for confounding variables such as pacifiers, collective day-care and parental occupation.

The results were the same after multivariate analysis (conditional logistic regression) as the ones found after multivariate analysis carried out on the entire study population.

We also carried two additional studies about the most frequent infections found in the group of children considered infected (sensitivity study): bronchiolitis and gastro-enteritis Regarding gastro-enteritis, there was a strong association between not being BF and infections from the age of 3 months and above (Table 5) with current BF and current EBF that protected from gastroenteritis even in multivariate analysis. When it came to bronchiolitis and aged matched comparisons (Table 6), BF protected children under the age of 9 and 12 months (but not below the age of 3 or 6 months). However, when the results were adjusted for six variables, BF wasn't confirmed as a protective factor due to the strong association between these confounding factors and bronchiolitis.

**Table 5:** Comparison between children affected by gastroenteritis and non-infected children or group B (*n* = 67 vs. 497) - Multivariate analysis (multiple logistic regression).

Type of food^a^	Children with gastroenteritis	Group B	Crude OR	IC 95%	*p*	Adjusted OR	IC 95% ^a^	*p*
	*N* (%)	*N* (%)	No adjustment			Adjustment on six variables		
Age <3-months (<13-weeks-old)	*N* = 16	*N* = 226						
Current BF or weaned BF vs. FD	5 (31.3)	117 (51.8)	0.42	0.13–1.25	0.11	0.41	0.12–1.36	0.14
Current BF vs. weaned BF + FD	2 (12.5)	91 (40.5)	0.21	0.05–0.95	0.027	0.20	0.04–0.96	0.045
Current EBF vs. others	0	64 (28.3)	--	--	0.013	--	--	--
Age < 6-months (< 26-weeks-old)	*N* = 32	*N* = 331						
Current BF or weaned BF vs. FD	9 (28.1)	168 (50.7)	0.38	0.17–0.84	0.013	0.39	0.16–0.94	0.035
Current BF vs. weaned BF + FD	4 (12.5)	121 (36.5)	0.25	0.08–0.72	0.006	0.28	0.09–0.88	0.03
Current EBF vs. others	1 (3.1)	90 (27.2)	0.09	0.01–0.64	0.003	0.09	0.01–0.70	0.02
Age < 9-month (< 39-weeks-old)	*N* = 46	*N* = 411						
Current BF or weaned BF vs. FD	16 (34.8)	208 (50.6)	0.52	0.27–0.98	0.041	0.55	0.28–1.10	0.09
Current BF vs. weaned BF + FD	6 (13.0)	136 (33.1)	0.30	0.12–0.73	0.005	0.38	0.15–0.98	0.044
Current EBF vs. others	2 (4.3)	101 (24.6)	0.14	0.03–0.59	0.002	0.18	0.04–0.79	0.023
Age < 12-months	*N* = 66	*N* = 475						
Current BF or weaned BF vs. FD	28 (42.4)	243 (51.1)	0.70	0.42–1.18	0.18	0.74	0.42–1.30	0.29
Current BF vs. weaned BF + FD	7 (10.6)	150 (31.6)	0.26	0.11–0.58	0.0004	0.37	0.16–0.87	0.022
Current EBF vs. others	4 (6.1)	116 (24.4)	0.20	0.07–0.56	0.001	0.26	0.09–0.77	0.014

^a^ EBF, Exclusively breastfed; PBF, Partially breastfed; BF, Breastfed; FD, Formula diet.

^b^ Adjustment on the age of the child, the socio-professional category of the parents, the parents or sibling allergies,, the type of childcare (home vs. others), the use of a pacifier, the gender of the child

= *OR < 1 means that Group A children are breastfed less often*.

**Table 6:** Comparison of feeding method since birth between children with bronchiolitis and non-infected children (group B).

Type of food^a^	Children with Groupe B bronchiolitis		Crude OR	95%CI	*p*	aOR	95% CI	*p*	aOR^b^	95% CI	*p*
	*N* (%)	*N* (%)	No adjusment			Adjustment on the age			Adjustment on 6 variables^b^		
Age <3-months	*N* = 58	*N* = 226									
Current BF or weaned BF vs. FD	33 (56.9)	117 (51.8)	1.21	0.67–2.16	0.53	1.19	0.66–2.15	0.56	1.37	0.73–2.57	0.32
Current BF vs. weaned BF or FD	20 (34.5)	91 (40.3)	0.77	0.42–1.41	0.39	0.83	0.45–1.54	0.55	0.91	0.47–1.76	0.78
Current EBF vs. other	15 (25.9)	64 (28.3)	0.88	0.46–1.70	0.71	0.91	0.47–1.78	0.79	1.03	0.51–2.11	0.93
Age < 6 months	*N* = 109	*N* = 331									
Current BF or weaned BF vs. FD	55 (50.5)	168 (51.1)	0.98	0.63–1.50	0.91	1.03	0.66–1.61	0.89	1.11	0.69–1.77	0.67
Current BF vs. weaned BF or FD	28 (25.7)	121 (36.8)	0.59	0.37–0.96	0.03	0.68	0.41–1.12	0.13	0.75	0.44–1.29	0.30
Current EBF vs. other	23 (21.1)	90 (27.2)	0.72	0.42–1.20	0.21	0.76	0.44–1.29	0.30	0.88	0.50–1.56	0.67
Age < 9 months	*N* = 144	*N* = 411									
Current BF or weaned BF vs. FD	74 (51.4)	208 (51.1)	1.01	0.69–1.48	0.95	0.91	0.70–1.50	0.91	1.09	0.73–1.64	0.66
Current BF vs. weaned BF or FD	31 (21.5)	136 (33.4)	0.55	0.35–0.86	0.008	0.62	0.39–0.97	0.038	0.72	0.44–1.17	0.19
Current EBF vs. other	24 (16.7)	101 (24.6)	0.61	0.38–1.00	0.051	0.68	0.41–1.12	0.13	0.82	0.48–1.40	0.47
Age < 12 months	*N* = 168	*N* = 475									
Current BF or weaned BF vs. FD	87 (52.1)	243 (51.6)	1.02	0.72–1.45	0.91	1.01	0.71–1.45	0.20	1.10	0.76–1.59	0.63
Current BF vs. weaned BF or FD	35 (21.0)	150 (31.8)	0.57	0.37–0.86	0.008	0.61	0.39–0.93	0.023	0.72	0.45–1.13	0.15
Current EBF vs. other	27 (16.2)	116 (24.4)	0.59	0.38–0.95	0.027	0.63	0.39–0.99	0.049	0.75	0.46–1.24	0.26

^a^ EBF, Exclusively breastfed; PBF, Partially breastfed; BF, Breastfed; FD, Formula diet.

^b^ Adjustment on the age of the child, the socio-professional category of the parents, the parents or sibling allergies,, the type of childcare (home vs. others), the use of a pacifier, the gender of the child.

*= OR < 1 means that Group A children are breastfed less often.*

In order to reveal a potential association between feeding habits and the severity of infections, especially bronchiolitis, we compared the hospitalisation rate as well as the Wang bronchiolitis severity score of the children from the group that were BF with the ones that weren't. Breastfeeding didn't reduce the severity. Regardless of the patient's age, there was no significant relationship between BF and other types of infections such as sore throat, laryngitis and rhino pharyngitis.

## Discussion

This study showed that, in France, a developed country, being exclusively BF is a protective factor against respiratory infections, gastroenteritis and otitis This protection was apparent for children exclusively BF or partially BF for more than 6 months, whereas breastfeeding for less than 6 months or stopping prematurely didn't appear to show any benefit preventing children from the infectious diseases that we looked at in this study. The protection was mainly against gastroenteritis, for children over the age of 3 months, and lesser so, against bronchiolitis. Moreover, this study showed that other factors could influence the occurrence of infectious diseases, such as pacifiers, the profession occupied by the child's parents and collective childcare. When a wider range of infectious diseases was analysed, no protective effect was shown, suggesting that BF only protects against broncho pulmonary infections, otitis and gastroenteritis.

The strengths of our study stemmed from the fact that it was multi-centric, with five paediatric emergency departments participating, allowing us to obtain a very diverse population sample, particularly socially. The substantial amount of patients included, gathering a wide range of data about potentially protective factors, three sensitivity studies as well as taking the age of the patients into account were also aspects that added to the strength of our work. Based on previous studies, we used the methodology recommended by Bauchner (13) in order to limit potential confounding factors.

The weaknesses shouldn't jeopardise the conclusions that can be drawn from our study. Patients were included during the autumn and winter months which are when rates of infections peak in children under the age of one. The patients included were all done so after being brought to the emergency department by their parents, regardless of whether they had symptoms that were compatible with an infection or not. The fact that no outpatients were included is a weakness. This method of inclusion could explain the over-representation of higher socio-professional categories in larger towns, which are more likely to BF but also occupy a full time job and use collective childcare, leading to multiple confounding factors. There could also be a statistic weakness in our study because there were a large number of statistical tests carried out; however, OR were far from 1 and highly significant which means the results and conclusions aren't in doubt.

This study confirms recent observations made in developed countries, BF protects against infections, more specifically respiratory tract infections (9–11, 14–17) and gastrointestinal infections (18, 19) with significant aOR between 0.45 and 0.70 but also infectious diseases as a whole (12, 20). This association has already been confirmed thanks to studies carried out in developed countries (21), for children that were BF for at least 6 months, with an OR of 0.57 (95% IC 0.44–0.75), as well as in studies carried out in developing countries, children being more frequently exclusively breastfed and for longer but confounding factors were often neglected (22, 23). Lastly, other factors, such as diet diversification (early or not), parental tobacco consumption and family or personal history of allergies, are not directly responsible for the occurrence of respiratory tract and gastrointestinal infections.

From a biological standpoint, the protection we found in our study could be explained by multiple mechanisms, both passive and active (24–26). Breast milk contains immunoglobulins, mostly secretory IgA, that are pathogen-specific in response to numerous enteric and respiratory infectious agents that the mother came across during the perinatal period. These immunoglobulins can prevent pathogen translocation in the gastrointestinal tract, neutralise toxins or other infectious agents. It also contains other bioactive factors (cytokines, chemokines, growth factors, hormones, lactoferrins …) capable of inhibiting inflammation, increasing the production of specific antibodies, facilitating the differentiation and growth of B lymphocytes leading to a better recognition of microorganisms. Oligosaccharides, which are also found in breast milk, are soluble complex carbohydrates that act as prebiotics, allowing certain beneficial strains of bacteria to develop, such as *Bifidobacterium infantis,* in the infant's gastrointestinal tract, therefore protecting the infant from the development of pathogenic bacteria. They also have direct antibacterial effects and inhibit pathogens from binding to the intestinal epithelium thanks to their « receptor decoys ». Oligosaccharides also modulate immune responses, enabling T lymphocytes to produce a balanced ratio of Th1/Th2 cytokines.

Breastfed children possess a different intestinal microbiome when compared to children fed using formulas, more stable, less diverse and containing more bacteria. Gene expression in the gastrointestinal tract after birth is influenced by BF, genes are expressed differently depending on whether the child is BF or fed with formula, regulating proliferation, differentiation and barrier function of intestinal epithelia.

Regarding children partially breastfed, their microbiome is more similar to formula fed children than exclusively BF, although it varies depending on the delivery method (27). However, weaning, rather than the introduction of solid food, seems to be the main factor behind developing an adult microbiome (28).

Regardless of the type of milk, physiological mechanisms protect from middle ear infections. Indeed, the suction needed for bottle feeding can create negative retro tympanum pressure.

In our study, BF seemed very much more protective against gastroenteritis than respiratory tract infections. We could hypothesize that this difference could be explained by the effect of oligosaccharides and secretory IgA on the gastrointestinal tract's mucosal surface. The fact that BF is a protective factor against gastroenteritis but not bronchiolitis, after multivariate analysis, shows that collective childcare or pacifiers have an effect on the risk of developing respiratory tract infections that is superior to the protective effect of BF. Furthermore, we weren't able to prove that breastfeeding had a protective effect when it came to the severity of the infection.

Our study shows that using pacifiers was strongly associated to the occurrence of infections, particularly respiratory tract infections. This association has rarely been the subject of studies and scientific publications, a study carried out in 1999 showed a link between using pacifiers and wheezing [OR = 1.23 (95% CI, 1.08–1.42; *p* < 0.05)] and gastro-enteritis [OR = 1.44 (95% CI, 1.18–1.75; *p* < 0.0001)] (29). Currently, there are no formal French recommendations about the use of pacifiers; however, the WHO advises against using pacifiers for breastfed children (30). There are diverging opinions regarding pacifiers and sudden infant death syndrome (31, 32), the interactions between pacifiers and the duration of breastfeeding being shortened are complex (33). A recent meta-analysis did not show a link between the pacifier and the duration of breastfeeding at 4 months and 6 months (for term newborns) (34). As sudden infant death syndrome occurs between 1 and 6 months of age, and the protective effect of BF only becomes apparent after 6 months, it would seem legitimate to recommend that pacifiers should stop being used after the age of 6 months.

As well as protecting against infections, studies have confirmed that BF protects against developing diabetes, obesity (35), asthma (36), eczema (8, 35, 37) and premature tooth decay (38). Breastfeeding is also beneficial for cognitive development (39, 40)with long-lasting effects (41). Despite all the beneficial effects of BF and the WHO's recommendation of exclusively BF for at least 6 months, few women, in France, BF for this long.

In our study, that included 5 hospitals in western France, only 18% of infants were still BF at the age of 6 months, the median duration of BF was 15 weeks. These results were similar to those published recently in France, 25% were breastfed at the age of 6 months and the median duration of BF was 15 weeks (42). Factors associated with BF not being initiated or stopped prematurely are well documented in the literature. In order to promote and favour BF in France, information about BF could be personalised and given earlier (before birth). The information given out could help to change the perception of BF and support specific couples that are less prone to BF or pursue breastfeeding (young mothers, little or no professional qualifications, low income, overweight). Adjusting work schedules as well as prolonging maternity leave could be envisaged; even though returning to work when deciding whether to pursue breastfeeding or not seems like a minor factor in the decision (42).

## Conclusion

This study shows that, in a developed country like France, despite the prevalence of BF being low, it protects against respiratory, gastrointestinal and ear infections as a whole, from the age of 6 to 12 months. This was the case for exclusively BF children and, to a lesser extent, for partially breastfed children. We took into account the many potentially confounding factors. The fact that there are complex interactions between BF and respiratory infections, as well as other factors that potentially favour infections such as pacifiers and collective childcare make for a difficult analysis, although BF is undoubtedly a protective factor in itself, adding to all the other medium and long term advantages. Amongst the information given out to the mother and the parents, breastfeeding, ideally exclusively, for at least the first 6 months, should be recommended. Actions should be carried out to help facilitate BF for children in collective childcare (expressing breast milk at mother's work place) and progressively stopping the use of pacifiers.

## Data Availability

The raw data supporting the conclusions of this article will be made available by the authors, without undue reservation.
